# Development of a text message intervention designed to promote safe contact lens wear

**DOI:** 10.1111/opo.13538

**Published:** 2025-06-13

**Authors:** Adam B. Samuels, Lisa J. Keay, Kate E. Faasse, Nicole A. Carnt

**Affiliations:** ^1^ School of Optometry and Vision Science, Faculty of Medicine and Health UNSW Sydney Sydney New South Wales Australia; ^2^ School of Psychology, Faculty of Science UNSW Sydney Sydney New South Wales Australia

**Keywords:** behaviour change, co‐design, contact lens compliance, contact lens safety, health promotion, text message

## Abstract

**Introduction:**

Some contact lens wearers demonstrate poor compliance with hygiene behaviours which increase their risk of corneal infection. Text message interventions for behaviour change can provide support and education in healthcare domains. This study reports on the co‐design of a text message intervention to target hygiene compliance, user satisfaction and discontinuation in contact lens wear.

**Methods:**

In phase 1, draft messages were composed using contact lens compliance advice from peak bodies, which was then optimised for persuasion using behaviour change theory. Phase 2 involved consultation with Patient Advocates (3), Health Psychology Experts (5) and Eyecare Practitioners (11), who rated messages (Likert 1–6) on readability, appropriateness, behaviour change and provided comments. Lay contact lens wearers participated in focus groups (2–4 per group) and provided feedback on relevance, comprehension and likely behaviour change. Phase 3 assessed messages and modified for readability (Flesh‐Kincaid). Phase 4 created and pilot tested (*n* = 5 users) text message sequences.

**Results:**

Phase 1 created 95 messages. In Phase 2, ratings (1–6) of readability (*M* = 5.4, *SD* = 0.5), appropriateness (*M* = 5.3 *SD* = 0.6) and likelihood to change behaviour (*M* = 5.0, *SD* = 0.6) combined with free text comments led to the modification of 59/95 (62%) messages, including the deletion of five messages. Focus group participants (5 groups, *n* = 14) suggested engagement, educational content and simplification. Most (62/90, 69%) messages were modified, four removed and two new messages proposed. In Phase 3, 88% were assessed as *fairly easy* or better. Post‐modification, all messages were *fairly easy*, mean readability 82.1 (range: 73.7–91.8, *SD* = 5.8). Phase 4 created 17 sequences of text messages and pilot testing established the process for replies, opt‐outs and modified time‐zone delivery.

**Conclusion:**

Co‐designing and evaluating text messages was feasible, resulting in a library of 88 optimised text messages formed into semi‐personalised sequences.

**Key points**

- The co‐design process is outlined for creating a library of text messages that target hygiene compliance, satisfaction and discontinuation in contact lens wear.
- In cases of contact lens‐related infection, non‐compliance with wear and care advice is a consistent risk factor.
- The library of text messages, optimised for health literacy and changing behaviour, is ready to be tested with patients.

## INTRODUCTION

Although eyecare practitioners (ECPs) usually provide wear and care compliance advice to contact lens (CL) wearers,^1^ many wearers still demonstrate non‐compliance with this advice, estimated at 40%–99% depending on the population and criteria.^2^, ^3^ Non‐compliant behaviours such as poor hand and lens hygiene are associated with an increased risk of microbial keratitis (MK)^4^ (estimated annualised incidence of 4 cases per 10,000),^5^ adverse events^6^ and CL‐related discomfort.^7^ Substantial vision loss of more than two lines of visual acuity occurs in an estimated 10%–14% of CL‐related MK cases.^8^ Given the widespread use of CLs^9^ and the potential consequences of non‐compliance, poor CL hygiene is a public health concern.

Contact lens discontinuation can occur when there has been an adverse event. However, the most frequently reported reason for CL discontinuation is discomfort in existing wearers^10^ and poor vision in neophyte CL wearers^11^ who demonstrate a higher risk of discontinuation, particularly during the first 2 months of CL wear.^12^ A recent systematic review suggested CL discontinuation occurs in approximately 25% of wearers over a 2‐ to 3‐year period.^13^ This rate of discontinuation of CL use can be thought of as a resource burden (expenditure of administrative and clinical time) on ECPs prescribing CLs.^14^

Public health promotion campaigns have been developed to enhance CL compliance at a population level; however, outcome measurement is challenging.^2^ Since 2020, the only published experimental interventions shown to enhance CL compliance are targeted education within a survey^15^ and an infographic ‘no water’ sticker,^16^ resulting in improvements in specific populations (those who have experienced a red‐eye adverse event) and specific behaviours (water exposure in reusable soft lens wearers), respectively. There is a lack of evidence‐based interventions designed to target overall compliance enhancement in multiple modalities of CL wear. Enhancing CL compliance by providing bite size education, reminders in the form of visually attractive messages and notifications via mobile phones have been suggested.^17^

Mobile phones are ubiquitous, personal, almost always carried and accessible to CL wearers. Market research reported that mobile phone users check their devices an average of seven times per hour, text messages are opened 90% of the time and 60% are read within 1–5 min of receipt.^18^ Text message interventions (TMIs) are increasing in popularity, and there is evidence that they can generate small positive effects in health behaviour change.^19^ TMIs are engaging,^20^ cost‐effective,^21^ can be automated to reach a large number of people and personalised to specific delivery times when support is most needed.^22^ In research, text messages have been utilised to obtain real‐time comfort and dry eye data from CL wearers,^23^ and commonly in clinical practice as CL appointment reminders. There is some evidence that a TMI for new CL wearers may reduce discontinuation in a 3‐month pre‐post study^24^; however, there are no published reports of TMIs designed for enhancing compliance with CL wear and care advice.

In studies that reported the development of TMIs for health behaviour change, there is evidence of consistency within the developmental processes,^25^, ^26^ such as the frequent use of behaviour change techniques (BCTs) incorporated into the design of TMIs to enact and optimise the effects.^27^ BCTs can be thought of as the ‘active psychological ingredients’ of an intervention.^28^ Instead of providing education or a reminder with a simple intervention, a BCT can provide strategies such as specific goal setting, help with intention formation and provide information about the approval of others, thereby encouraging behaviour change. In addition, the involvement of co‐designers (to generate content, provide feedback and pretesting) can offer advantages such as enhanced interactions between providers and consumers and enriched consumer engagement and experiences.^29^ The use of co‐design can mitigate the dominance of the healthcare practitioner mindset and instead involve patients more in the decision‐making processes. Few health promotion TMIs have addressed health literacy (the ability to understand and use information to promote health) in their development.^30^ Both health literacy and general literacy are important social determinants of health, and increasing health literacy is linked to improved ocular health outcomes,^31^ correlating positively with higher levels of CL compliance.^32^

The objective of this study is to describe the procedures and outcomes of a systematic process leading to a library and sequence of text messages for contact lens wearers. The aim is to design a TMI targeting CL compliance, satisfaction and discontinuation with the use of co‐design, behavioural theory and health literacy optimisation.

## METHODS AND RESULTS

**FIGURE 1:**
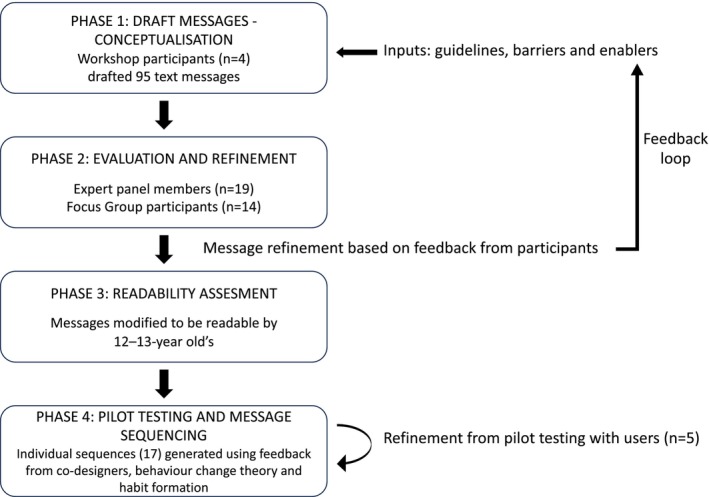
Programme design steps.

As this study involved an iterative four‐phase process, the corresponding results are presented after each method phase. Ethical approval was obtained from the University of New South Wales (UNSW) Sydney Human Research Ethics Approval Panel C: Behavioural Sciences (File 3463). All participants provided written online consent. The development of the *Programme Design Steps* (Figure 1) was adapted from previous successful TMI studies^33^, ^34^ and published developmental frameworks.^26^

### Phase 1 method: Draft messages–conceptualisation

The aim of Phase 1 was to draft a library of messages to provide education, motivation, support and reminders with the aim of enhancing CL compliance and wearer satisfaction and reducing discontinuation. Online CL educational guidelines (in English) from global optometry and CL organisations were verified to be evidence‐based, where appropriate, and similar content was synthesised and deleted. Websites accessed (in March 2021) belonged to the Cornea and Contact Lens Society of Australia, British Contact Lens Association, Optometry Australia, the College of Optometrists (UK), Centers for Disease Control and Prevention (US), American Optometric Association and the Food and Drug Administration (US). Compliance information assessed pertained to daily disposable, frequent replacement soft lenses and rigid gas permeable (RGP) lenses. Excluded content related to recommended overnight use (including orthokeratology), specialty lenses (such as sclerals) and for therapeutic use (drug delivery and myopia control).

BCTs are active psychological ingredients of an intervention, which can be used to change behaviour. Appropriate BCTs were selected from a taxonomy,^35^ which included *Provide Instruction*, *Prompt Intention Formation* and *Prompt Specific Goal Setting*, and incorporated within the structure of each message to optimise the effect (*see* Table 1). Each BCT was underpinned by theoretical frameworks that propose changing behaviour by providing education, skills, motivation, intention and goal setting, combined with approval from others.^36^, ^37^

**TABLE 1:** Examples of text messages including incorporated behaviour change techniques, theoretical frameworks and source content with website.

Message theme	Behaviour change technique^a^ (theoretical framework)	Example text message	Source content (website)
Administration		Hello (*Name* ^b^)! Welcome to TextClean. Thank you for participating. If you wish to opt‐out please reply ‘STOP’. TextClean, UNSW	N/A
Compliance New wearers	Provide instruction (*Social cognitive theory*) Model or demonstrate the behaviour (*Social cognitive theory*)	Hey (*Name*)! learning how to put lenses on and remove them is not always easy. There is more than one correct way to do it – if you need a refresher try these links: Putting them on – click here.^c^ Removing them – click here. TextClean,^d^ UNSW	Always wash and dry your hands prior to handling your lenses. Rub, rinse and store your lenses in the recommended solution before and after each use (except single‐use lenses, which should be discarded after each wear). Clean the lens case with solution, wipe with a clean tissue then air‐dry after each use by placing the case and lids face down on a tissue. Apply the same lens first to avoid mixing them up. Check the lens is not inside out or damaged before applying. Handle carefully to avoid damaging the lens. Apply your lenses before putting on make‐up. (BCLA)
COMPLIANCE Daily disposable wearers	Prompt Information about others approval (*Theory of reasoned action model, theory of planned behaviour, Information‐motivation behavioural skills model*) Prompt specific goal setting (*Control theory*)	Hi (*Name*)! Did you know that the happiest CL wearers always use fresh lenses every day… …and always take them out before sleeping too! TextClean, UNSW	Daily disposables are designed only for single‐use and must be discarded after each wear. These lenses should be used strictly on a daily wear basis and are not intended or approved for re‐use or for overnight wear. Daily disposables have a low risk of problems when used correctly. (BCLA)
Compliance Water exposure	Prompt specific goal setting (*Control theory*) Agree on behavioural contract (*Operant conditioning*) Provide instruction (*Social cognitive theory*)	(*Name*), did you know that water contains germs that are dangerous for the eyes? Can you keep all water away from your lenses from now on? Click here for more information (https://www.cdc.gov/contactlenses/water‐and‐contact‐lenses.html) TextClean, UNSW	You should never use tap water in any area of your lens care, including rinsing the lenses and the lens case. Do not attempt to make your own homemade saline or CL solutions. Also, make sure your hands are completely dry before handling your lenses. (AOA)
Discontinuation	Prompt Information about others approval (*Theory of reasoned action model, theory of planned behaviour, Information‐motivation behavioural skills model*) Provide instruction (*Social cognitive theory*) Provide information about a behaviour health link (*Information‐motivation behavioural skills model*)	(*Name*), are you on your screen all day? Dry eyes can be grumpy eyes. Most people who take regular breaks, blink often and use lubricant drops, will notice improvement. TextClean, UNSW	Air‐conditioning, heating or windy days may exacerbate lens dehydration and often using an unpreserved (unit dose) lubricant may be of assistance – check with your optometrist which type is most suitable for your lens type. (OA)
Satisfaction	Provide general encouragement and Prompt self‐talk (*Social cognitive theory*)	Hi (*Name*), did you know that research has shown that we often feel better about our appearance when we are not wearing our glasses. How good do you feel when you are wearing your contacts? TextClean, UNSW	Compared to wearing glasses, switching to CLs reported significant improvements in the areas of perceived appearance, participation in activities and satisfaction with vision correction (CDC)

Abbreviations: AOA, American Optometric Association; BCLA, British Contact Lens Association; CDC, Centers for Disease Control and Prevention; OA, Optometry Australia; UNSW, University of New South Wales.

^a^ The BCTs taxonomy is further detailed in Appendix S1.

^b^ (*Name*) is a place holder for the auto‐population of the participant's name.

^c^ ‘Click here’ refers to embedded weblinks to educational videos within the text message.

^d^ ‘TextClean UNSW’ is the signoff signature included for source credibility.

In addition, the messages were designed to be positively framed (describing the benefits of good compliance as opposed to the negative consequences of poor compliance). Evidence suggests that framed messages (compared to non‐framed statements) and positive framing (compared to negative framing) may be more effective and preferred.^38^, ^39^, ^40^, ^41^, ^42^, ^43^

Further design considerations were based on a synthesis of communications frameworks and recommendations from previously successful TMI interventional trials. Accounting for a range of health literacy levels, the messages were designed to be brief (less than 400 characters), written in a non‐clinical, informal style^44^ (without abbreviations) and included user‐friendly tips and links to websites and videos.^25^, ^26^ Messages were designed to be compelling, semi‐personalised (using the recipients' name) and credible with the use of the academic institute of the investigators, ‘UNSW Sydney’ signature.

To create the text message library, a message co‐design workshop was held with three academic ECPs (AS,* SY,† SK‡) and one health psychology expert (MV§). To direct the message drafting, all workshop designers were provided with synthesised content from global optometry and CL websites and a brief introduction to the taxonomy and application of BCTs. Examples from the text message library are displayed in Table 1.

### Phase 1 results: Draft messages—conceptualisation

A library of 95 messages was created that provided education, motivation, support and reminders in the areas of CL compliance (72/95), discontinuation prevention (9/95) and wearer satisfaction (7/95). In addition, there were seven administration messages created. The messages were created as credible, compelling, unoffensive, simple and unambiguous. All field notes were maintained, documenting the process of content development.

### Phase 2 method: Evaluation and refinement

#### Expert panel members (*n* = 19)

Three prominent patient advocates in the field of CL hygiene and infection communication, five health psychology experts (PhD candidate level or above) and 11 ECPs with experience in CL practice (5 or more years) were recruited using existing professional networks. Each panel member reviewed sequential sets of draft messages (*n* = 20) via an online survey Qualtrics XM (qualtrics.com). Messages were scored with a six‐point Likert scale; *completely disagree* (1) to *completely agree* (6) on readability, appropriate content and likely impact on changing the behaviour of CL wearers. Panel members could provide free text comments for each message.

#### Focus groups of contact lens wearers (*n* = 14)

A mixed methods recruitment strategy recruited CL wearers. Partner ECPs at a community practice (Specsavers Eastgardens, New South Wales) distributed flyers to CL wearers and advertising was placed on the investigators' (AS) social media (Facebook, Twitter and LinkedIn). Those with professional expertise in the CL field such as eyecare professionals and practice staff were excluded from participating. QR codes on flyers and hyperlinks in online advertisements directed participants to the study screening and consent. A sample size guide of a minimum of four focus groups was deemed appropriate.^45^ Five focus groups were conducted online. Each focus group, comprising two to four participants, was provided with a sequential set of 15 messages (previously modified by the expert panel). Focus group participants were asked specific questions relating to each message: relevance, ease of understanding, length and likely behaviour change potential. The process of deductive thematic analysis using predetermined themes was conducted and illustrative comments supporting each theme were aggregated. Feedback was video recorded on Teams Classic (Microsoft, microsoft.com) and transcribed verbatim using NVivo 12 (Lumivero, lumivero.com).

### Phase 2 results: Evaluation and refinement

#### Expert panel

There were 300 reviews (each draft message reviewed three to four times) and a total of 96 free‐text comments. Overall, message readability, content and impact on behaviour change were rated highly as ‘strongly agree’ (see Table 2). Review scores of less than 4.0 or reviewer comments resulted in refinement or deletion from the library.

**TABLE 2:** The expert panel review of draft messages.

Expert panel review	Domain	Review rating, mean (SD)
Likert rating, 1 (completely disagree), 2 (disagree), 3 (somewhat disagree), 4 (somewhat agree), 5 (agree), 6 (completely agree)	Readability	5.4 (0.5)
	Appropriateness of content	5.3 (0.6)
	Likely impact on changing the behaviour of CL wearers	5.0 (0.6)

Expert panel process	Action	Number of draft messages
	Keep	26/76 (34%)
	Refine	45/76 (59%)
	Delete	5/76 (7%)

Final library outcomes		
76 draft messages plus duplicate (12) and administrative messages (7)	Messages kept	29/95 (31%)
	Messages refined	54/95 (57%)
	Messages deleted	5/95 (5%)
	Administrative messages	7/95 (7%)

Abbreviations: CL, contact lenses; SD, Standard deviation.

Seven messages containing administrative content only were not presented to the review panel. In addition, 12 messages with content written for monthly wearers were duplicated for use with two‐weekly lens wearers and were therefore only presented once to the review panel. In total, there were 76/95 draft messages presented to the expert panel. Final determination of each individual message was based on a synthesis of reviewer ratings and free text comments and resulted in the options of *Keep, Refine or Delete*. More than half of all the draft messages were refined (57%, 54/95) or deleted (5%, 5/95) based on this process.

The three patient advocates in the field of CL hygiene communication (who had all previously suffered CL‐related *Acanthamoeba* keratitis) suggested using loss‐framing language in 18 message reviews, such as ‘clean your lens case otherwise you may get an infection and lose your sight’. For these disagreements, the views of the majority and the basis for positively framed messages were prioritised,^38^, ^39^, ^40^, ^41^, ^42^, ^43^ while incorporating the gravity of potential sight loss.

#### Focus groups

Recruited CL wearers (9/14 female) used daily disposables (5/14), monthly reusables (6/14), two‐weekly reusables (2/14) and RGPs (1/14). Age ranged from 18 to over 55 years (median age bracket of 36 to 45 years). Contact lens wearing experience ranged from 1 year to over 15 years. CL wearers assessed the relevance, ease of understanding, length and likely behaviour change potential of each draft message. Most (62/90, 69%) of the draft messages were modified in response to this focus group feedback. Four messages were deleted due to poor understanding and low relevance. Two new messages were proposed and accepted into the library pertaining to saline solution and travel bottles of CL solution. Thematic analysis of all comments resulted in recommendations (presented in Table 3) which were applied to the appropriate messages within the library.

**TABLE 3:** Focus group thematic analysis resulted in the following number of specific comments per message.

Theme	Improve readability	Reduce message length	Increase engagement	Increase educational content
Number of comments	45/90	11/90	7/90	5/90

### Phase 3 method: Readability assessment

The text message library was assessed for ease of reading with the Flesch–Kincaid readability algorithm.^46^ Reading Ease scores between 90 and 100 are classed as *very easy* and <10 as *extremely difficult*. The aim was to ensure that all messages could be assessed as *fairly easy* (readable by 12‐ to 13‐year‐old's), equivalent to a Reading Ease score of between 70 and 80. Messages that did not conform to this criterion were modified to improve readability by removing or replacing large and unnecessary words and by shortening sentences.

### Phase 3 results: Readability assessment

Initial assessment resulted in 77/88 (88%) of the messages being classed as ‘*fairly easy’* (reading ease score of between 70 and 80), readable by a 12‐ to 13‐year‐olds. The remaining messages 11/88 (12%) were assessed as being ‘standard difficulty’, readable by a 13‐ to 15‐year‐old. Poor scoring messages (Reading Ease score <70) were modified (*n* = 10). Post‐modification, final assessment of the modified message library assessed all messages as ‘fairly easy’, readable by a 12‐ to 13‐year old with a mean Reading Ease score of 82.1 (range: 73.7–91.8, standard deviation: 5.8).

### Phase 4 method: Pilot testing and message sequencing

The content and delivery of the TMIs underwent pretesting with recipients (*n* = 5) who were CL wearers and known to the research team. Over a testing period of 10 days, the delivery of the text messages to recipients on different mobile networks and time zones within Australia was investigated. The TMIs were sent using the online automated platform Kudosity (kudosity.com, previously known as Burst SMS). Verification was sought on the receipt of the messages across different time zones, the ability to reply, to opt‐out, reply receipt and logistics of stopping and restarting the sequence.

The message sequencing was developed by synthesising feedback from co‐designers, pilot testing, published literature on behaviour change theory, habit formation applied to TMI design and trial implementation (Appendix S2). The messages were ordered in sequences to promote specifically the formation of new CL healthy habits.^47^

### Phase 4 results: Pilot testing and message sequencing

Logistical pilot testing by recipients confirmed that messages were received as programmed within a few minutes of being sent. Recipients also verified the ability to send replies and to opt‐out from receiving messages. Verification of recipient replies and sequence stop/restart was confirmed. One message was received incorrectly at 06:13 h in the time zone of Western Australia, resulting in delivery programming refinement. The final library consisted of 88 messages, from which 17 text message sequences were developed (mean messages per sequence 69, range: 56–76). Message sequences varied in content depending on the type of lens wear (such as daily disposable or frequent replacement lenses) and experience of the participant (new wearer or seasoned wearer). Design considerations (Appendix S2) resulted in the placement of educational messages early in the sequence to raise awareness of appropriate hygiene. Mid‐sequence messages encouraged participants to act on the education and to form resolutions or agreements. At the latter stage of the sequence, messages reminded and prompted participants to review their new habits.

## DISCUSSION

This study presents the outcomes of a co‐design framework, leading to the creation of a library of text messages targeting CL compliance, satisfaction and discontinuation. This is the first text message library to be designed for CL compliance enhancement. We are unaware of any previous reports of this application and could find no reference to it in a computerised PubMed search from 2003 to 2024. The systematic process of incorporating co‐design, behaviour change theory and health literacy optimisation into the messages has been documented in this study. Messages were rated highly for readability, appropriateness and likely behaviour change impact. Most messages were modified due to feedback from co‐designers, and the recommended themes of engagement, message clarity and educational content from the CL wearer focus groups were incorporated throughout. Finally, logistical pilot testing and the sequence creation verified that the messages were ready for efficacy testing.

The co‐design included opinions from those who have examined CL wearers in practice, currently wear CLs, have experienced loss of vision due to CL‐related infection and understand the psychology of health conditions. These different inputs ensured the messages would be suitable for a broad range of CL wearers. The patient advocates expressed preferences for the use of threatening language referencing the loss of vision from infection. For the final message bank, the opinions of the patient advocates were balanced with inputs such as the evidence supporting the use of positively framed messages for consumers,^42^ and consideration that fear‐based messaging may not be appropriate for unidirectional health communication, as evidence suggests it may contribute to discontinuation in successful CL wearers.^48^ Aside from this example, where there were differences in message ratings between CL wearers and ECPs, the wearers input took precedence, ensuring that their perspectives remained central throughout the design process. This approach stands in contrast to conventional methods of ECP‐derived health message promotion content,^49^ with potential improvements in consumer engagement, service delivery and health outcomes.^50^, ^51^

TMIs are gaining traction as effective health behaviour change strategies; however, there is little evidence of published TMIs specifically for ocular health promotion. Two studies reported similar TMI developmental processes in diabetic retinopathy education^52^ and in myopia control education.^53^ These did not include health literacy optimisation with readability assessment nor educational weblinks. Text messages incorporating educational weblinks have been used to promote health literacy in priority eye diseases successfully.^54^

Almost half of all Australian adults have both low health literacy^55^ and general literacy equivalent to 15–16 years of age or below,^56^ and most online patient education ocular health advice is at a readability level beyond the Australian average.^57^ With increasing numbers of children wearing CLs, there was value in optimising for general literacy (readable by 12‐ to 13‐year‐olds) and for health literacy (bite‐size information and educational hyperlinks).

A limitation of this study was the small sample size of the CL wearer focus groups, and that each message was reviewed by one focus group only (2–4 participants). Notably, new wearers (wearing CLs for less than 2 months) and the age bracket 25–34 years did not have their opinions represented, and this may have impacted the final message content. Second, the messages were written only in English, and future research to adapt the library for diverse cultures and languages will be required. In addition, the message library was created with current evidence, and it will be necessary to update the library with emerging evidence. A full clinical trial evaluating the efficacy of the TMIs has been published,^58^ including the feasibility and acceptability of the messages (with measures such as frequency, content, language, delivery and cost).

In conclusion, the systematic process incorporating co‐design with CL wearers, ECPs, health psychology experts and patient advocates has been documented. A library of 88 text messages was created, optimised for health literacy and behaviour change and designed to target CL compliance, satisfaction and discontinuation.

## AUTHOR CONTRIBUTIONS

**Adam B. Samuels:** Conceptualization (lead); formal analysis (lead); investigation (lead); methodology (lead); project administration (lead); writing – original draft (lead); writing – review and editing (lead). **Lisa J. Keay:** Conceptualization (equal); supervision (equal); writing – review and editing (equal). **Kate E. Faasse:** Conceptualization (equal); supervision (equal); writing – review and editing (equal). **Nicole A. Carnt:** Conceptualization (equal); supervision (equal); writing – review and editing (equal).

## FUNDING INFORMATION

NC was awarded a UNSW Scientia Fellowship and AS was supported by a PhD Scientia Scholarship. This research received no specific grant from any funding agency in the public, commercial or not‐for‐profit sectors.

## CONFLICT OF INTEREST STATEMENT

All authors attest that they meet the current ICMJE criteria for authorship. AS, KF and LK indicate no financial disclosures or conflicts of interest. NC has previously received an educational grant from Alcon, honoraria from the British Contact lens Association (UK) and Optometry Virtually Connected and travel grants from Standards Australia and Australian Vision Convention (QLD/NT).

## CONSENT

All participants provided written online consent.

## Supporting information


Appendix S1.

Appendix S2.

## Footnotes

* AS is an ECP and PhD Candidate.

† SY is an ECP and PhD Candidate researching CL compliance.

‡ SK is an ECP and PhD Candidate researching dry eye.

§ MV is a physiotherapist and PhD Candidate researching rituals in health psychology.

## References

[1] Alonso S , Yela S , Cardona G . Are patients sufficiently informed about contact lens Wear and care? Optom Vis Sci. 2022;99:853–858. 36441991 10.1097/OPX.0000000000001964

[2] Cope JR , Collier SA , Rao MM , Chalmers R , Mitchell GL , Richdale K , et al. Contact lens wearer demographics and risk behaviors for contact lens‐related eye infections—United States, 2014. Morb Mortal Wkly Rep. 2015;64:865–870. 10.15585/mmwr.mm6432a2 PMC5779588 26292204

[3] Bui T , Cavanagh H , Robertson D . Patient compliance during contact lens wear: perceptions, awareness and behavior. Eye Contact Lens. 2010;36:334–339. 20935569 10.1097/ICL.0b013e3181f579f7 PMC3148150

[4] Stapleton F . Contact lens‐related corneal infection in Australia. Clin Exp Optom. 2020;103:408–417. 32363626 10.1111/cxo.13082

[5] Szczotka‐Flynn LB , Shovlin JP , Schnider CM , Caffery BE , Alfonso EC , Carnt NA , et al. American Academy of Optometry microbial keratitis think tank. Optom Vis Sci. 2021;98:182–198. 33771951 10.1097/OPX.0000000000001664 PMC8075116

[6] Yee A , Walsh K , Schulze M , Jones L . The impact of patient behaviour and care system compliance on reusable soft contact lens complications. Cont Lens Anterior Eye. 2021;44:101432. 10.1016/j.clae.2021.02.018 33678542

[7] Dumbleton K , Woods C , Jones L , Richter D , Fonn D . Comfort and vision with silicone hydrogel lenses: effect of compliance. Optom Vis Sci. 2010;87:421–425. 20386353 10.1097/OPX.0b013e3181d95aea

[8] Stapleton F , Keay L , Edwards K , Naduvilath T , Dart JKG , Brian G , et al. The incidence of contact lens–related microbial keratitis in Australia. Ophthalmology. 2008;115:1655–1662. 18538404 10.1016/j.ophtha.2008.04.002

[9] Dumbleton K , Caffery B , Dogru M , Hickson‐Curran S , Kern J , Kojima T , et al. The TFOS international workshop on contact lens discomfort: report of the subcommittee on epidemiology. Invest Ophthalmol Vis Sci. 2013;54:TFOS20–TFOS36. 24058130 10.1167/iovs.13-13125

[10] Richdale K , Sinnott LT , Skadahl E , Nichols JJ . Frequency of and factors associated with contact lens dissatisfaction and discontinuation. Cornea. 2007;26:168–174. 17251807 10.1097/01.ico.0000248382.32143.86

[11] Sulley A , Young G , Hunt C . Prospective evaluation of new contact lens wearer retention rates. Cont Lens Anterior Eye. 2018;41:S4. 10.1016/j.clae.2016.10.002

[12] Sulley A , Young G , Hunt C . Factors in the success of new contact lens wearers. Cont Lens Anterior Eye. 2017;40:15–24. 27818113 10.1016/j.clae.2016.10.002

[13] Jones L , Efron N , Bandamwar K , Barnett M , Jacobs DS , Jalbert I , et al. TFOS lifestyle: impact of contact lenses on the ocular surface. Ocul Surf. 2023;29:175–219. 37149139 10.1016/j.jtos.2023.04.010

[14] Nichols JJ , Willcox MDP , Bron AJ , Belmonte C , Ciolino JB , Craig JP , et al. The TFOS international workshop on contact lens discomfort: executive summary. Invest Ophthalmol Vis Sci. 2013;54:TFOS7–TFOS13. 24058135 10.1167/iovs.13-13212 PMC4686219

[15] Lam D , Wagner H , Zimmerman AB , Rosner B , Kinoshita B , Mickles C , et al. Change in risk score and behaviors of soft contact lens wearers after targeted patient education. Eye & Contact Lens: Science & Clinical Practice. 2022;48:347–354. 10.1097/ICL.0000000000000900 35580482

[16] Arshad M , Carnt N , Tan J , Stapleton F . Compliance behaviour change in contact lens wearers: a randomised controlled trial. Eye. 2020;35:88–95. 10.1038/s41433-020-1015-9 PMC8027657 32546749

[17] Cardona G , Alonso S , Yela S . Compliance versus risk awareness with contact lens storage case hygiene and replacement. Optom Vis Sci. 2022;99:449–454. 35165235 10.1097/OPX.0000000000001881

[18] Dobrilova T . 35+ must‐know SMS marketing statistics. [updated in 2025], Available from: https://techjury.net/industry‐analysis/sms‐marketing‐insights/. Accessed March, 2021.

[19] Armanasco AA , Miller YD , Fjeldsoe BS , Marshall AL . Preventive health behavior change text message interventions: a meta‐analysis. Am J Prev Med. 2017;52:391–402. 28073656 10.1016/j.amepre.2016.10.042

[20] Hall AK , Cole‐Lewis H , Bernhardt JM . Mobile text messaging for health: a systematic review of reviews. Annu Rev Public Health. 2015;36:393–415. 25785892 10.1146/annurev-publhealth-031914-122855 PMC4406229

[21] Burn E , Nghiem S , Jan S , Redfern J , Rodgers A , Thiagalingam A , et al. Cost‐effectiveness of a text message programme for the prevention of recurrent cardiovascular events. Heart. 2017;103:893–894. 10.1136/heartjnl-2016-310195 28235776

[22] Nahum‐Shani I , Smith SN , Spring BJ , Collins LM , Witkiewitz K , Tewari A , et al. Just‐in‐time adaptive interventions (JITAIs) in mobile health: key components and design principles for ongoing health behavior support. Ann Behav Med. 2018;52. 10.1007/s12160-016-9830-8 PMC5364076 27663578

[23] Santodomingo‐Rubido J , Barrado‐Navascués E , Rubido‐Crespo M‐J . Ocular surface comfort during the day assessed by instant reporting in different types of contact and non–contact lens wearers. Eye Contact Lens. 2010;36:96–100. 20145542 10.1097/ICL.0b013e3181d1d5a5

[24] Patel K . The impact on new contact lens wearer retention after introduction of a patient support tool. Cont Lens Anterior Eye. 2022;45:101683. 10.1016/j.clae.2022.101683

[25] Ricci‐Cabello I , Bobrow K , Islam SMS , Chow CK , Maddison R , Whittaker R , et al. Examining development processes for text messaging interventions to prevent cardiovascular disease: systematic literature review. JMIR Mhealth Uhealth. 2019;7:e12191. 10.2196/12191 30924790 PMC6460311

[26] Abroms LC , Whittaker R , Free C , Van Alstyne JM , Schindler‐Ruwisch JM . Developing and pretesting a text messaging program for health behavior change: recommended steps. JMIR Mhealth Uhealth. 2015;3:e107. 10.2196/mhealth.4917 26690917 PMC4704898

[27] Free C , Phillips G , Galli L , Watson L , Felix L , Edwards P , et al. The effectiveness of mobile‐health technology‐based health behaviour change or disease management interventions for health care consumers: a systematic review. PLoS Med. 2013;10:e1001362. 10.1371/journal.pmed.1001362 23349621 PMC3548655

[28] Michie S , Richardson M , Johnston M , Abraham C , Francis J , Hardeman W , et al. The behavior change technique taxonomy (v1) of 93 hierarchically clustered techniques: building an international consensus for the reporting of behavior change interventions. Ann Behav Med. 2013;46:81–95. 23512568 10.1007/s12160-013-9486-6

[29] Steen M , Manschot M , De Koning N . Benefits of co‐design in service design projects. Int J Des. 2011;5:53–60.

[30] Cheng C , Beauchamp A , Elsworth GR , Osborne RH . Applying the electronic health literacy lens: systematic review of electronic health interventions targeted at socially disadvantaged groups. J Med Internet Res. 2020;22:e18476. 10.2196/18476 32788144 PMC7453328

[31] Capó H , Edmond JC , Alabiad CR , Ross AG , Williams BK , Briceño CA . The importance of health literacy in addressing eye health and eye care disparities. Ophthalmology. 2022;129:e137–e145. 36058736 10.1016/j.ophtha.2022.06.034

[32] Dagtekin G , Unsal A , Caliskan Pala S , Ocal EE , Arslantas D , Simsek T . Contact lens usage and health literacy among Turkish adults. Marmara Med J. 2022;35:67–72.

[33] Redfern J , Thiagalingam A , Jan S , Whittaker R , Hackett M , Mooney J , et al. Development of a set of mobile phone text messages designed for prevention of recurrent cardiovascular events. Eur J Prev Cardiol. 2014;21:492–499. 22605787 10.1177/2047487312449416

[34] Singleton A , Raeside R , Partridge SR , Hayes M , Maka K , Hyun KK , et al. Co‐designing a lifestyle‐focused text message intervention for women after breast cancer treatment: mixed methods study. J Med Internet Res. 2021;23:e27076. 10.2196/27076 34125072 PMC8240797

[35] Abraham C , Michie S . A taxonomy of behavior change techniques used in interventions. Health Psychol. 2008;27:379–387. 18624603 10.1037/0278-6133.27.3.379

[36] Bandura A . Social foundations of thought and action: a social cognitive theory. Englewood Cliffs, New Jersey: Prentice‐Hall, Inc; 1986. p. xiii.

[37] Fishbein M . A reasoned action approach to health promotion. Med Decis Making. 2008; 28:834–844. 19015289 10.1177/0272989X08326092 PMC2603050

[38] Wansink B , Pope L . When do gain‐framed health messages work better than fear appeals? Nutr Rev. 2015;73:4–11. 10.1093/nutrit/nuu010 26024053

[39] Ratcliff CL , Jensen JD , Scherr CL , Krakow M , Crossley K . Loss/gain framing, dose and reactance: a message experiment. Risk Anal. 2019;39:2640–2652. 31361043 10.1111/risa.13379

[40] Williams J , Saken M , Gough S , Hing W . The effects of message framing characteristics on physical activity education: a systematic review. Cogent Med 2019;6:1666619. 10.1080/2331205X.2019.1666619

[41] Gao R , Guo H , Li F , Liu Y , Shen M , Xu L , et al. The effects of health behaviours and beliefs based on message framing among patients with chronic diseases: a systematic review. BMJ Open. 2022;12:e055329. 10.1136/bmjopen-2021-055329 PMC8739424 34992117

[42] Redfern J , Santo K , Coorey G , Thakkar J , Hackett M , Thiagalingam A , et al. Factors influencing engagement, perceived usefulness and behavioral mechanisms associated with a text message support program. PLoS One. 2016;11:e0163929. 10.1371/journal.pone.0163929 27741244 PMC5065147

[43] Livi S , Zeri F , Baroni R . Health beliefs affect the correct replacement of daily disposable contact lenses: predicting compliance with the health belief model and the theory of planned behaviour. Cont Lens Anterior Eye. 2017;40:25–32. 27671981 10.1016/j.clae.2016.09.003

[44] World Health Organization . WHO strategic communications framework for effective communications. Ginebra: WHO; 2017.

[45] Hennink MM , Kaiser BN , Weber MB . What influences saturation? Estimating sample sizes in focus group research. Qual Health Res. 2019;29:1483–1496. 30628545 10.1177/1049732318821692 PMC6635912

[46] Kincaid JP , Fishburne RP Jr , Rogers RL , Chissom BS . Derivation of new readability formulas (automated readability index, fog count and flesch reading ease formula) for navy enlisted personnel. Millington, TN: Naval Technical Training Command Millington Tennessee Research Branch; 1975.

[47] Lally P , Gardner B . Promoting habit formation. Health Psychol Rev. 2013;7:S137–S158.

[48] Bennett ES , Stulc S , Bassi CJ , Schnider CM , Morgan BW , Henry VA , et al. Effect of patient personality profile and verbal presentation on successful rigid contact lens adaptation, satisfaction and compliance. Optom Vis Sci. 1998;75:500–505. 9703038 10.1097/00006324-199807000-00018

[49] McMonnies CW . Improving contact lens compliance by explaining the benefits of compliant procedures. Cont Lens Anterior Eye. 2011;34:249–252. 21798791 10.1016/j.clae.2011.06.006

[50] Sumner J , Ng CWT , Teo KEL , Peh ALT , Lim YW . Co‐designing care for multimorbidity: a systematic review. BMC Med. 2024;22:58. 10.1186/s12916-024-03263-9 38321495 PMC10848537

[51] Silvola S , Restelli U , Bonfanti M , Croce D . Co‐design as enabling factor for patient‐centred healthcare: a bibliometric literature review. ClinicoEconomics and Outcomes Research. 2023;15:333–347. 37220481 10.2147/CEOR.S403243 PMC10200122

[52] Umaefulam VO , Premkumar K . Development of text messages for Mobile health education to promote diabetic retinopathy awareness and eye care behavior among indigenous women. Cham, Switzerland: Springer International Publishing; 2018. p. 107–118.

[53] Keel S , Govender‐Poonsamy P , Cieza A , Faal H , Flitcroft I , Gifford K , et al. The WHO‐ITU MyopiaEd programme: a digital message programme targeting education on myopia and its prevention. Front Public Health. 2022;10:881889. 10.3389/fpubh.2022.881889 35692340 PMC9177978

[54] Sharma IP , Chaudhry M , Sharma D , Kaiti R . Mobile health intervention for promotion of eye health literacy. PLOS Global Public Health. 2021;1:e0000025. 10.1371/journal.pgph.0000025 36962088 PMC10021255

[55] Safety ACo, Care QiH . Health literacy: taking action to improve safety and quality. Sydney, Australia: Australian Commission on Safety and Quality in Health Care; 2015.

[56] https://www.abs.gov.au/statistics/people/education/programme‐international‐assessment‐adult‐competencies‐australia/latest‐release. Accessed 28 May, 2025.

[57] Huang G , Fang CH , Agarwal N , Bhagat N , Eloy JA , Langer PD . Assessment of online patient education materials from major ophthalmologic associations. JAMA Ophthalmology. 2015;133:449–454. 25654639 10.1001/jamaophthalmol.2014.6104

[58] Samuels A , Keay L , Faasse K , Carnt N . Effect of text messages designed to change contact lens compliance: a randomised controlled trial. Cont Lens Anterior Eye. 2025;48:102341. 10.1016/j.clae.2024.102341 39603861

