# Supplementary material for: Development of a text message intervention designed to promote safe contact lens wear
Source: Ophthalmic Physiol Opt. 2025 Jun 13;45(6):1261–9. doi: 10.1111/opo.13538 (PMC12357223; doi:10.1111/opo.13538)
Supplement: Supplementary file 1 — Appendix S1. [file OPO-45-1261-s001.docx]

**Behaviour Change Technique taxonomy (adapted from Abraham and Michie, 2008)**

|  | **Behaviour Change Technique (Theoretical Framework)** | **Definition** | **Application to CL Compliance** |
| --- | --- | --- | --- |
| 1 | **Provide information about behaviour- health link. (IMB)** | General information about behavioural risk, for example, susceptibility to poor health outcomes or mortality risk in relation to the behaviour | Provide Information on Health Risks: Educate CL wearers about the risks associated with non-compliance, such as increased susceptibility to MK, and other CL related complications. |
| 2 | **Provide information on consequences. (TRA, TPB, SCogT, IMB)** | Information about the benefits and costs of action or inaction, focusing on what will happen if the person does or does not perform the behaviour | Discuss Benefits and Costs: Clearly explain the benefits of complying to CL practices (for example, maintaining good eye health and comfort) and the costs of non-compliance, such as having to cease CL wear and the potential for serious eye conditions |
| 3 | **Provide information about others’ approval. (TRA, TPB, IMB)** | Information about what others think about the person’s behaviour and whether others will approve or disapprove of any proposed behaviour change | Highlight Social Norms and Approval: Inform CL wearers about the positive social norms regarding proper CL care, emphasising that most CL users follow the guidelines and that eye care professionals strongly approve of compliance to maintain eye health. |
| 4 | **Prompt intention formation. (TRA, TPB, SCogT, IMB)** | Encouraging the person to decide to act or set a general goal, for example, to make a behavioural resolution such as “I will take more exercise next week‘ | Encourage Goal Setting: Motivate CL wearers to set specific goals related to CL care, such as committing to cleaning CLs every day or replacing them as recommended. |
| 5 | **Prompt barrier identification. (SCogT)** | Identify barriers to performing the behaviour and plan ways of overcoming them | Identify Barriers and Solutions: Work with CL wearers to identify barriers to compliance, such as forgetting to replace CLs or lacking proper cleaning supplies, and develop strategies to overcome these obstacles, such as setting reminders or keeping supplies readily available. |
| 6 | **Provide general encouragement. (SCogT)** | Praising or rewarding the person for effort or performance without this being contingent on specified behaviours or standards of performance | Provide Praise: Praise CL wearers for their efforts in improving their CL care routines, regardless of whether they have met specific compliance targets yet, to encourage continued effort. |
| 7 | **Set graded tasks. (SCogT)** | Set easy tasks, and increase difficulty until target behaviour is performed. | Start with Easy Tasks: Begin with simple tasks, such as remembering to wash hands before handling CLs, and gradually introduce more complex behaviours |
| 8 | **Provide instruction. (SCogT)** | Telling the person how to perform a behaviour and/or preparatory behaviours | Give Clear Instructions: Provide clear and detailed instructions on how to properly clean and store CLs, emphasising each step in the process to ensure proper technique. |
| 9 | **Model or demonstrate the behaviour(SCogT).** | An expert shows the person how to correctly perform a behaviour, for example, in class or on video | Demonstrate Proper Techniques: Have an eye care professional demonstrate the correct way to handle and clean CLs using through instructional videos or infographics. |
| 10 | **Prompt specific goal setting. (CT)** | Involves detailed planning of what the person will do, including a definition of the behaviour specifying frequency, intensity, or duration and specification of at least one context, that is, where, when, how, or with whom | Create a Detailed Care Plan: Help CL wearers develop a specific plan that outlines when and how to perform each aspect of CL care, including the frequency of CL replacement and cleaning procedures. |
| 11 | **Prompt review of behavioural goals. (CT)** | Review and/or reconsideration of previously set goals or intentions | Review and Reevaluate Goals: Regularly review CL wearers' progress towards their compliance goals to ensure continued improvement and compliance to guidelines. |
| 12 | **Prompt self-monitoring of behaviour. (CT)** | The person is asked to keep a record of specified behaviour (s) (e.g., in a diary) | Encourage Behaviour Tracking: Ask CL wearers to keep a diary of their CL care practices, noting each time they clean their CLs, replace them, or visit their eye care provider. |
| 13 | **Provide feedback on performance. (CT)** | Providing data about recorded behaviour or evaluating performance in relation to a set standard or others’ performance, i.e., the person received feedback on their behaviour | Provide Feedback on Compliance: Offer feedback on CL wearers' performance by comparing their behaviour with recommended practices, highlighting areas of success and opportunities for improvement. |
| 14 | **Provide contingent rewards. (OC)** | Praise, encouragement, or material rewards that are explicitly linked to the achievement of specified behaviours | Use Contingent Rewards: Offer praise and encouragement as rewards, for achieving defined compliance milestones, like consistently washing hands before CL handling or removing CLs before a shower |
| 15 | **Teach to use prompts or cues. (OC)** | Teach the person to identify environmental cues that can be used to remind them to perform a behaviour, including times of day or elements of contexts. | Use Environmental Cues: Help CL wearers identify environmental cues that can remind them to perform necessary care tasks, such as placing CL solution next to their toothbrush to prompt cleaning before bed. |
| 16 | **Agree on behavioural contract. (OC)** | Agreement (e.g., signing) of a contract specifying behaviour to be performed so that there is a written record of the person’s resolution witnessed by another | Establish a Behavioural Contract: Encourage CL wearers to agree to behavioural contracts as suggested by the intervention. Encourage CL wearers to tell friends or family members to reinforce accountability. |
| 17 | **Prompt practice. (OC)** | Prompt the person to rehearse and repeat the behaviour or preparatory behaviours | Prompt Rehearsal of Care Routine: Encourage CL wearers to think about their CL care routine, to reinforce correct practices and ensure they become habitual. |
| 18 | **Use follow-up prompts.** | Contacting the person again after the main part of the intervention is complete | Conduct Follow-Ups: Schedule follow-up interventions or after the initial intervention to check on CL wearers’ progress and provide additional support or guidance as needed. |
| 19 | **Provide opportunities for social comparison. (SCompT)** | Facilitate observation of nonexpert others’ performance for example, in a group class or using video or case study | Facilitate Peer Observation: Discuss how others practice proper CL care, with the use of video demonstrations with fellow CL users. |
| 20 | **Plan social support or social change. (social support theories)** | Prompting consideration of how others could change their behaviour to offer the person help or (instrumental) social support, including “buddy” systems and/or providing social support | Encourage Social Support: Suggest ways CL wearers can involve family or friends in their CL care routine, such as having a "CL buddy" remind them to replace their CLs or providing moral support for maintaining compliance. |
| 21 | **Prompt identification as a role model** | Indicating how the person may be an example to others and influence their behaviour or provide an opportunity for the person to set a good example | Promote Role Modelling: Highlight CL wearers who consistently follow recommended CL care practices as role models for others, encouraging them to set a good example and influence peers. |
| 22 | **Prompt self-talk.** | Encourage use of self-instruction and self-encouragement (aloud or silently) to support action | Teach Self-Instructions: Encourage CL wearers to use positive self-talk to reinforce proper CL care practices, such as mentally reminding themselves to clean their CLs before inserting them. |
| 23 | **Relapse prevention. (relapse prevention therapy)** | Following initial change, help identify situations likely to result in readopting risk behaviours or failure to maintain new behaviours and help the person plan to avoid or manage these situations | Plan for Relapse Prevention: Help CL wearers identify situations that might lead to lapses in compliance, such as travel or busy schedules, and develop strategies to prevent these lapses from occurring. |
| 24 | **Stress management (stress theories)** | May involve a variety of specific techniques (e.g., progressive relaxation) that do not target the behaviour but seek to reduce anxiety and stress | Introduce Stress Reduction Techniques: Teach stress-reduction techniques, such as mindfulness or relaxation exercises, to help CL wearers manage anxiety or stress that may interfere with their ability to comply with proper CL care practices. |
| 25 | **Motivational interviewing** | Prompting the person to provide self-motivating statements and evaluations of their own behaviour to minimize resistance to change | Prompt Self-Motivating Statements: Encourage CL wearers to make self-motivating statements and evaluate their own behaviour, such as reminding themselves of the benefits of proper CL care and the personal commitment to eye health. |
| 26 | **Time management** | Helping the person make time for the behaviour (e.g., to fit it into a daily schedule) | Help Schedule Behaviour: Assist wearers in finding ways to incorporate CL care into their daily routines, such as setting specific times for cleaning and replacing CLs to make compliance more habitual and convenient |

Theoretical frameworks underpinning the BCTs are IMB = information-motivation- behavioural skills model; TRA = theory of reasoned action; TPB = theory of planned behaviour; SCogT = social-cognitive theory; CT = control theory; OC = operant conditioning.
