# Supplementary material for: Development of a text message intervention designed to promote safe contact lens wear
Source: Ophthalmic Physiol Opt. 2025 Jun 13;45(6):1261–9. doi: 10.1111/opo.13538 (PMC12357223; doi:10.1111/opo.13538)
Supplement: Supplementary file 2 — Appendix S2. [file OPO-45-1261-s002.docx]

**The evidence and theory underpinning the design of each text message sequence**

| **Item** | | **Evidence** | **Sequence Design Considerations** |
| --- | --- | --- | --- |
| **Length of program** |  | Evidence suggests a 6-12 month program period is linked to effect enhancement^25^, and creating habit change can take from 18-254 days^62^ | The program was designed to run for 6-months |
| **Timing**  **of message delivery** | Random times | Random times may be effective for habit disruption^25,28^  Conforming to polite SMS etiquette protocols has been suggested ^28,32,33^ | Messages were sent at random times during the hours between 10:00 and 18:00, allowing for time zone differences.  Messages were excluded from being sent at weekends |
| **Timing**  **of message delivery** | Specific times | For certain behaviours that were time sensitive, targeted Just-in-time-adaptive-interventions were planned for additional impact^29^ | To remind wearers to remove lenses before sleep, targeted *overnight wear* themed messages sent between 21:00 and 22:00 |
| **Frequency of message delivery** |  | Some evidence suggests that multiple messages per day^63^.  while most available evidence points to a decreasing frequency over time^25,64^ | A decreasing frequency of messages over time was scheduled: 4 times per week for the first three months and 2 messages per week for the final three months |
| **Personalised** |  | Although there is limited evidence for personalisation increasing effect^25,26,65,66^, personalisation may be important for acceptability and engagement which may impact effectivity^39,40^ | The messages were semi-personalised using each participants’ name for example:  *“Hi Alice, did you know that water and contacts don’t mix?”* |
| **Targeted** | New Wearers | New CL wearers are risk of discontinuing lenses particularly during the first 2 months of wear with vision and handling techniques being important factors^13^ | Messages designed to *demonstrate behaviour*^^[[1]](#footnote-1)^^ specifically for CL insertion and removal techniques for new wearers were scheduled so that they had two messages with links to a video demonstration within the first 2 months |
| **Targeted** | Cosmetics | It is clear that certain types of cosmetics have detrimental effect on the ocular surface^67^ and can impact CL parameters and performance^68^, however there is lack of evidence to guide exact protocol^69^ | Contact lens wearers who used cosmetics around the eyes received tailored messages to remind them to use caution with application of cosmetics and creams. |
| **Tailoring** |  | Although there is limited evidence for tailoring increasing intervention effect^25,26,65,66^, tailoring may be important for acceptability and engagement which may impact effectivity^39,40^ | Tailoring the message by inserting the participants preferred *activity* into a pre-formatted message such as: “*Hi Sam, do you want to keep using your contacts for -playing footy? Lens wearers all agree that it's vital to wash your hands…* |
| **Habit formation** | Overview | Meta analysis of behaviour change interventions suggest that habits are more effective than knowledge and beliefs^70^ | Structure the sequence of text messages to help create good compliance habits using the theoretical 4-step approach^61^ |
| **Habit formation** | Step 1 | Step 1 - Understanding the behaviour and its consequences | Messages early in the sequence to raise awareness in the habit change process  Educational messages that used *behaviour-health links*^[[2]](#footnote-2)^ such as informing of risks of overnight wear and water exposure and their *consequences*^[[3]](#footnote-3)^. |
| **Habit formation** | Step 2  and  Step 3 | Step 2 - Intention to change  Step 3 - Using self-efficacy(agency) to commit to action  (Steps 2 and 3 were initiated once the risks and correct technique were understood and the rapport and relationship had been built) | Messages encouraged participants *to make a resolution*^[[4]](#footnote-4)^ to take action along with *agreement or a deal*^[[5]](#footnote-5)^ for example,  *Do you take your clothes off before a shower? - you should remove your contacts too! Research shows that for healthy eyes, keep contacts away from water. If you are not doing this already, can we plan to try this for the next 2 weeks?* |
| **Habit formation** | Step 4 | Step 4 - Repetition and follow up | Messages prompted participants to review their new *habit resolutions*^[[6]](#footnote-6)^ and agreements such as:  “*A gentle reminder about our plan to remove contacts before having a shower! How are you progressing?”* |
| **Habit formation** | Stage 4 | Messages that congratulated participants and provided feedback on their expected improvement | Messages *providing feedback*^[[7]](#footnote-7)^ on successful behaviour change such as “*keep it up and give yourself a pat on the back for looking after your eye health and vision*” |
| **Habit formation** | Stage 4 | Habit change is not linear^71^, and participants don’t always read every message. | Important messages were repeated at later stages in the sequence |

1. Behaviour change technique used: Model or demonstrating the behaviour [↑](#footnote-ref-1)
2. Behaviour change technique used: Provide information about behaviour- health link [↑](#footnote-ref-2)
3. Behaviour change technique used: Provide information on consequences [↑](#footnote-ref-3)
4. Behaviour change technique used: Prompt intention formation [↑](#footnote-ref-4)
5. Behaviour change technique used: Agree on behavioural contract [↑](#footnote-ref-5)
6. Behaviour change technique used: Prompt review of behavioural goals [↑](#footnote-ref-6)
7. Behaviour change technique used: Provide feedback on performance [↑](#footnote-ref-7)
